# Supplementary material for: Challenges and opportunities for high-quality battery production at scale
Source: Nat Commun. 2025 Jan 12;16:611. doi: 10.1038/s41467-025-55861-7 (PMC11725600; doi:10.1038/s41467-025-55861-7)
Supplement: Supplementary file 1 — Supplementary Information [file 41467_2025_55861_MOESM1_ESM.pdf]

Supplementary Information for

Challenges and opportunities for high-quality battery production at scale

Peter M. Attia[1]*, Eric Moch[1], Patrick K. Herring[1]

1. Glimpse, 444 Somerville Avenue, Somerville, MA 02143, USA

***Corresponding author**: peter@glimp.se

Supplementary Figures

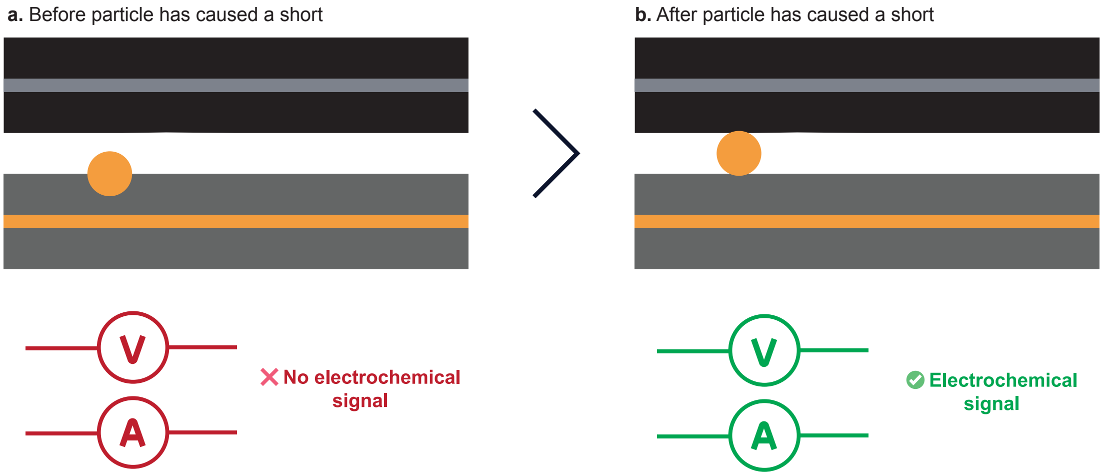

Supplementary Fig. 1 | Illustration of latent defects and their detectability via electrochemical signals. (a) Electrode stack with a copper particle on the surface of the negative electrode. Since the particle has not yet pierced the separator, electrochemical techniques cannot capture a signal. (b) Electrode stack with a copper particle that has pierced the separator and electronically connects the negative and positive electrodes. Only at this point is a signal of the internal short detectable via electrochemical characterization. Thus, by definition of a latent defect, electrochemical techniques will fail to detect these defects before they have activated. By extension, non-electrochemical techniques are required to catch latent defects before they activate.

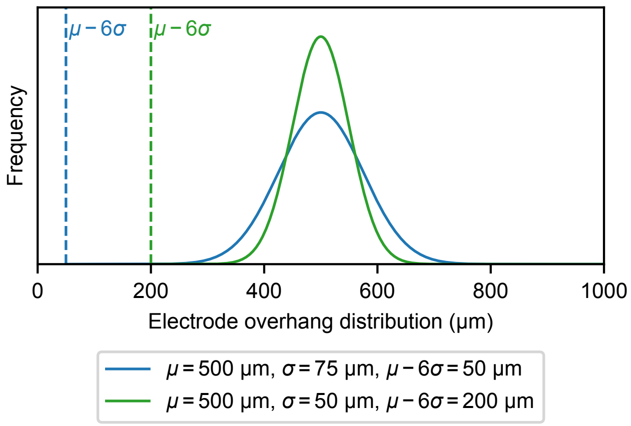

Supplementary Fig. 2 | Potential benefits of increased conformance on cell energy. The solid blue curve represents the overhang distribution for a population of cells. The distribution has a mean of 500 µm and a standard deviation of 75 µm (identical to Figure 5a). The solid green curve is identical except that the standard deviation is 50 µm. This reduction in standard deviation translates to a 150 µm difference in the six-sigma lower bound. For a cylindrical cell with an average positive electrode width of 64 mm, this additional 300 µm (150 µm for the top and bottom of the cell) would translate to an additional 0.5% of positive electrode width and thus an additional 0.5% energy. Accurate, high-throughput inspection is required to quantify these trends.

Supplementary Tables

Supplementary Table 1 | Statistical parameters used to create the data in Figure 5. Here, LSL refers to "lower specification limit", and USL refers to "upper specification limit". The parameters so that 0.5% of the cells would fall below the LSL in (a) and (e), and 0.1% of the cells would fall below the LSL in (b), (c), and (d). Note that in practice, the LSL and USL would likely be set as six-sigma limits (i.e., 50 μm to 950 μm) to yield a defective rate of 3.4 defects per million. The mean of 500 μm was obtained from the 500-cell batch scanned in Condon et al.[1]

Subplot	Mean (μm)	Standard deviation (μm)	LSL (μm)	USL (μm)
a. Original	500	75	305	800
b. Expand spec limits	500	75	268	800
c. Shift mean	537	75	305	800
d. Tighten distribution	500	60	305	800
e. Improve inspection	500	75	305	800

Supplementary Discussions

Supplementary Discussion 1 | Brief review of influential factors for battery failure

Cell design.—Nearly every aspect of cell design impacts cell lifetime and failure. The structure and composition of the active components (electrodes and electrolytes) is perhaps the most widely recognized factor for battery lifetime.[2–5] However, inactive components (binders, separators, tapes, foils, tabs, casings, seals, etc.) are, in our view, equally important determinants of battery lifetime and failure.[6–13] Furthermore, the interactions between battery geometry (form factor, size, etc.) and lifetime/failure are interesting yet poorly understood.[14,15,12] Finally, the battery formation protocol can be considered a subset of cell design and is also a significant lever for battery lifetime.[16–18]

Module/pack design.—OEMs can also influence battery lifetime and failure via module and pack design. One major interaction is the thermal management strategy of the battery pack, which will be a major determinant of the internal temperatures that the cells experience.[19–21] Of course, the module/pack mechanical design (i.e., cell spacing, inter-cell thermal insulation, etc.) is a major determinant of cell-level resistance to safety events.[22,23] In the case of pouch cells, the module/pack mechanical design also controls the stack pressure; excessive pressure, especially when combined with naturally-occurring electrochemomechnical swelling, can cause plating and thus performance degradation and perhaps internal shorting as well.[24,25] Recent reports indicate that even cell orientation (i.e., horizontal vs. vertical) can influence lifetime.[26,27] A final critical aspect is the pack-level electric design and the cell balancing strategy. These design choices determine the magnitude of, and the response to, voltage and/or current imbalance between cells or between modules.[21,28–30] If internal shorts are unable to be detected and/or remediated, the magnitude of the short could grow and potentially lead to thermal runaway.[31,32]

Cell operating limits.—Cells are often designed with specific operating limits for parameters such as cutoff voltages and charging rate. These specifications may be multidimensional matrices (e.g., maximum charging rate as a function of SOC and temperature). Widening these operating limits may unlock additional performance (e.g., range for cutoff voltages and fast charging times for charging rate) but often at the expense of lifetime.[33–36] Many of these operating limits impact the occurrence of functional failures and safety events (e.g., knees, internal shorting due to lithium plating, and thermal runaway due to overcharge).[29,33,36]

End-user behavior.—Within the specified cell operating limits, end-user behavior can significantly influence battery lifetime and failure. (Here, we use the term “end user” to refer to the ultimate user of the battery-powered product, since the term “customers” may include OEMs purchasing cells from a cell producer). Some well-established risk factors for battery lifetime include the number of full equivalent cycles, time spent at SOC extremes, and the extent of fast charging.[33,37] Given that EV customers range from office commuters in Houston to rideshare drivers in New York, a truly massive range of end-user behaviors must be considered with regards to battery failure.

External environment.—Battery failure is sensitive to the environmental conditions of operation. The most well-established environmental sensitivity is that of temperature: most batteries operate best within an optimal temperature range, outside of which the lifetime may be reduced.[33,38] Environmental mechanical conditions can also hasten battery failure; for instance, vibrations and shocks can cause functional failures[39], and violent mechanical events such as a crash can cause safety events[22]. Lastly, the chemical environment of batteries can cause additional degradation, as ambient humidity can introduce unwanted moisture and ambient salinity can cause corrosion—especially if a transport pathway from the environment to the cell internals exists (e.g., poor seal

life).[40,41] In general, the module- and pack-level controls will determine how sensitive cell lifetime and failure will be to the external environment.

We emphasize that the interactions between these factors can make both cell design and cell failure diagnosis inordinately complex. For all categories of battery failure, the root cause may be due to cell characteristics, end-user behavior, or a combination of both. For instance, a massively defective cell may fail under any reasonable use case; a nominally healthy cell may fail under aggressive use conditions outside of the manufacturer’s intent; and a moderately defective cell may fail when used in a moderately aggressive use case. In the latter scenario, attribution is difficult; more importantly, balancing these concerns in practice is even more so. The end result of this complexity is that the design and execution of a test suite that spans this multidimensional variability is no small undertaking.

Supplementary Discussion 2 | Testing details for Figure 7

All tests were performed on a BYD FC4680 cylindrical cell.

Ultrasound

The cell was inserted into EchoStat THETA system (produced by Liminal Insights, Inc.), which consists of a cell fixture, translation/rotation stage, ultrasonic transducers, dry coupling material, and an electronics chain. Measurements were contact-based and used through transmission. The resulting signal was processed to extract Amplitude RMS, which corresponds to degree of wetness (higher value tends to indicate higher electrolyte saturation). The scan acquisition time was three hours; Liminal suggested that this time could be significantly reduced with a multi-transducer pair system as opposed to the single transducer pair.

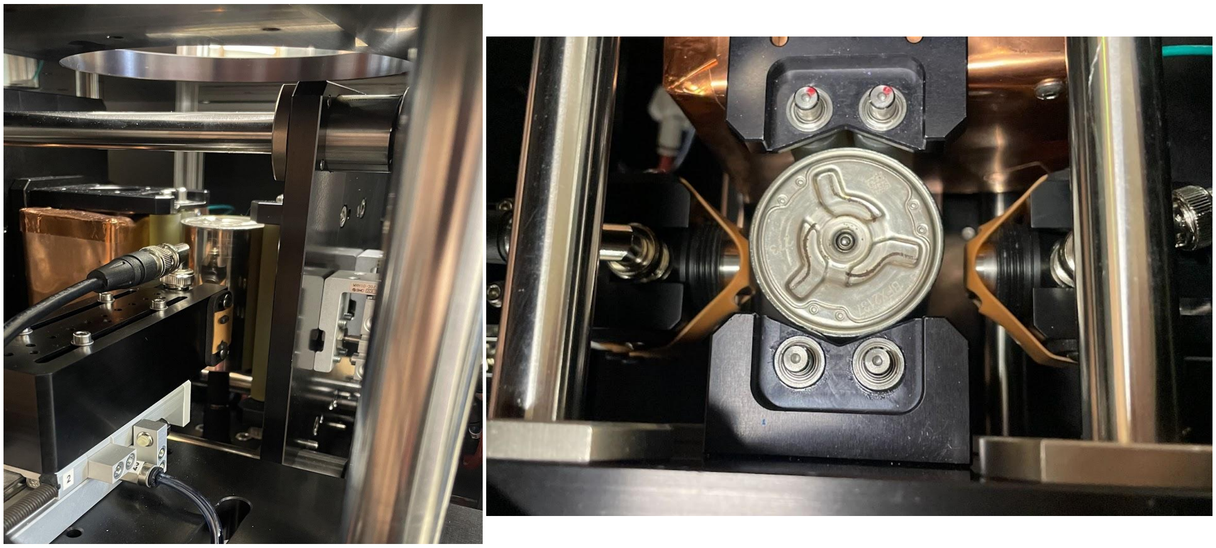

Photographs of the EchoStat THETA system (produced by Liminal Insights, Inc.).

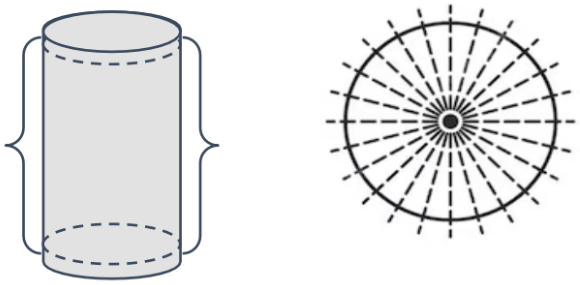

	Coverage & Resolution
**Z**	65 mm total, 3mm pitch
θ	360° total, 3° pitch
**No. of positions**	2280

Schematic and experimental details of the measurement positions. The system translates & rotates the cell to collect acoustic data at multiple positions. Then, acoustic features extracted from the signal are stitched together to form a sonograph.

<u>2D X-ray</u>

The cell was imaged using a Nikon XT H 225 ST 2X system equipped with a rotating anode source using Inspect-X version XT 6.12. The X-ray voltage was 210 kV and the power was 51.0 W. Three images were acquired: one of the full cell, one of the top overhang region, and one of the bottom overhang region. The pixel size was 33.7 μm for the full cell image and 17.5 μm for the two overhang images. The image acquisition time was 125 ms for all images. The brightness and contrast of the images were uniformly adjusted in the figure. Note that the horizontal line in the middle of the left image is a result of the fixture used to position the cell in the X-ray system.

3D X-ray

The cell was imaged using a Nikon XT H 225 ST 2X system equipped with a rotating anode source using Inspect-X version XT 6.12. The X-ray voltage was 210 kV and the power was 90.1 W. The voxel size was 33.7 μm, and the scan acquisition time was 73 seconds (note that we foresee many hardware and software levers to further reduce scan acquisition time). The images were processed through Glimpse's CT scan processing pipeline. The brightness and contrast of the images were uniformly adjusted in the figure.

Supplementary Discussion 3 | Cost-benefit analysis for inspection techniques

In this discussion, we estimate the cost per kWh of 2D X-ray as well as the cost per kWh of field failures. These estimates require several assumptions (not all of which have sources) but provide context into the cost-benefit analysis of inspection.

<u>Part 1: Estimated cost per kWh of 2D X-ray</u>

Assumptions:

- A production-grade 2D X-ray system costs around $350,000 for equipment and installation.
- This system can image 175 cylindrical cells per minute (ppm).[42]
- These cylindrical cells are 2170 cells (21 mm diameter, 70 mm height) with a nominal cell energy of 18 Wh.
- The cost of this equipment is amortized over 7 years.
- 10% of the initial cost ($35,000) is required every year for preventative maintenance, replacement parts, etc.
- A battery production facility runs 24 hours a day, 7 days a week, 365 days per year.

Calculations:

- The yearly cost is $350,000 / 7 years + $35,000/year = $85,000/year.
- In 1 year, (175 cells/minute) * (60 minutes/hour) * (24 hours/day) * (365 days/year) ≈ 92 million cells are imaged.
- The total energy of these ~92 million cells = ~92 million cells * 18 Wh/cell = 1.66 GWh/year = 1,660,000 kWh/year.
- The cost per kWh of 2D X-ray is thus $85,000 / 1,660,000 kWh = **$0.05/kWh**.

Part 2: Estimated cost per kWh of field failures

Assumptions:

- A typical field failure rate for EVs is 2.5%.[43]
    - Note that publicly-available data for this statistic is difficult to come by.
- Each battery pack costs $15,000 to replace.[44]
- Each battery pack has a nominal energy of 50 kWh.[45]
- The cost of decreased sales due to higher prices is excluded from this analysis.
- The costs of bad publicity, poor user experience, etc. are excluded from this analysis.

Calculations:

- All EVs must be sold for an additional 2.5% * $15,000 = $375 to cover the additional warranty costs for pack replacement.
- This cost translates to $375 / 50 kWh = **$7.50/kWh**.

Supplementary References

1. Condon, A. *et al.* A dataset of over one thousand computed tomography scans of battery cells. *Data Brief* **55**, 110614 (2024).
2. Birkl, C. R., Roberts, M. R., McTurk, E., Bruce, P. G. & Howey, D. A. Degradation diagnostics for lithium ion cells. *J. Power Sources* **341**, 373–386 (2017).
3. Edge, J. S. *et al.* Lithium ion battery degradation: what you need to know. *Phys. Chem. Chem. Phys.* **23**, 8200–8221 (2021).
4. Xu, K. Nonaqueous Liquid Electrolytes for Lithium-Based Rechargeable Batteries. *Chem. Rev.* **104**, 4303–4418 (2004).
5. Xu, K. Electrolytes and Interphases in Li-Ion Batteries and Beyond. *Chem. Rev.* **114**, 11503–11618 (2014).
6. Carter, R., Huhman, B., Love, C. T. & Zenyuk, I. V. X-ray computed tomography comparison of individual and parallel assembled commercial lithium iron phosphate batteries at end of life after high rate cycling. *J. Power Sources* **381**, 46–55 (2018).
7. Fear, C., Juarez-Robles, D., Jeevarajan, J. A. & Mukherjee, P. P. Elucidating Copper Dissolution Phenomenon in Li-Ion Cells under Overdischarge Extremes. *J. Electrochem. Soc.* **165**, A1639–A1647 (2018).
8. Lagadec, M. F., Zahn, R. & Wood, V. Characterization and performance evaluation of lithium-ion battery separators. *Nat. Energy* **4**, 16–25 (2019).
9. Zhang, X. & Wierzbicki, T. Characterization of plasticity and fracture of shell casing of lithium-ion cylindrical battery. *J. Power Sources* **280**, 47–56 (2015).
10. Lee, K.-J., Smith, K., Pesaran, A. & Kim, G.-H. Three dimensional thermal-, electrical-, and electrochemical-coupled model for cylindrical wound large format lithium-ion batteries. *J. Power Sources* **241**, 20–32 (2013).
11. Waldmann, T., Geramifard, G. & Wohlfahrt-Mehrens, M. Influence of current collecting tab design on thermal and electrochemical performance of cylindrical Lithium-ion cells during

high current discharge. *J. Energy Storage* **5**, 163–168 (2016).

12. Tranter, T. G., Timms, R., Shearing, P. R. & Brett, D. J. L. Communication—Prediction of Thermal Issues for Larger Format 4680 Cylindrical Cells and Their Mitigation with Enhanced Current Collection. *J. Electrochem. Soc.* **167**, 160544 (2020).
13. Adamson, A. *et al.* Improving lithium-ion cells by replacing polyethylene terephthalate jellyroll tape. *Nat. Mater.* **22**, 1380–1386 (2023).
14. Quinn, J. B., Waldmann, T., Richter, K., Kasper, M. & Wohlfahrt-Mehrens, M. Energy Density of Cylindrical Li-Ion Cells: A Comparison of Commercial 18650 to the 21700 Cells. *J. Electrochem. Soc.* **165**, A3284–A3291 (2018).
15. Kim, H.-K., Choi, J. H. & Lee, K.-J. A Numerical Study of the Effects of Cell Formats on the Cycle Life of Lithium Ion Batteries. *J. Electrochem. Soc.* **166**, A1769 (2019).
16. An, S. J. *et al.* The state of understanding of the lithium-ion-battery graphite solid electrolyte interphase (SEI) and its relationship to formation cycling. *Carbon* **105**, 52–76 (2016).
17. Attia, P. M., Harris, S. J. & Chueh, W. C. Benefits of Fast Battery Formation in a Model System. *J. Electrochem. Soc.* **168**, 050543 (2021).
18. Weng, A. *et al.* Predicting the impact of formation protocols on battery lifetime immediately after manufacturing. *Joule* **5**, 2971–2992 (2021).
19. Rothgang, S., Baumhofer, T. & Sauer, D. U. Diversion of Aging of Battery Cells in Automotive Systems. in *2014 IEEE Vehicle Power and Propulsion Conference (VPPC)* 1–6 (IEEE, Coimbra, Portugal, 2014). doi:10.1109/VPPC.2014.7007050.
20. Liu, X., Ai, W., Naylor Marlow, M., Patel, Y. & Wu, B. The effect of cell-to-cell variations and thermal gradients on the performance and degradation of lithium-ion battery packs. *Appl. Energy* **248**, 489–499 (2019).
21. Reniers, J. M. & Howey, D. A. Digital twin of a MWh-scale grid battery system for efficiency and degradation analysis. *Appl. Energy* **336**, 120774 (2023).
22. Feng, X., Ren, D., He, X. & Ouyang, M. Mitigating Thermal Runaway of Lithium-Ion

Batteries. *Joule* **4**, 743–770 (2020).

23. Lamb, J. & Jeevarajan, J. A. New developments in battery safety for large-scale systems. *MRS Bull.* **46**, 395–401 (2021).
24. Cannarella, J. & Arnold, C. B. Stress evolution and capacity fade in constrained lithium-ion pouch cells. *J. Power Sources* **245**, 745–751 (2014).
25. Bach, T. C. *et al.* Nonlinear aging of cylindrical lithium-ion cells linked to heterogeneous compression. *J. Energy Storage* **5**, 212–223 (2016).
26. Milojevic, Z. *et al.* Influence of orientation on ageing of large-size pouch lithium-ion batteries during electric vehicle life. *J. Power Sources* **506**, 230242 (2021).
27. Fordham, A. *et al.* Correlative non-destructive techniques to investigate aging and orientation effects in automotive Li-ion pouch cells. *Joule* **7**, 2622–2652 (2023).
28. Omariba, Z. B., Zhang, L. & Sun, D. Review of Battery Cell Balancing Methodologies for Optimizing Battery Pack Performance in Electric Vehicles. *IEEE Access* **7**, 129335–129352 (2019).
29. Dubarry, M., Devie, A. & Liaw, B. Y. Cell-balancing currents in parallel strings of a battery system. *J. Power Sources* **321**, 36–46 (2016).
30. Weng, A., Siegel, J. B. & Stefanopoulou, A. Differential voltage analysis for battery manufacturing process control. *Front. Energy Res.* **11**, (2023).
31. Zhang, G. *et al.* Internal short circuit mechanisms, experimental approaches and detection methods of lithium-ion batteries for electric vehicles: A review. *Renew. Sustain. Energy Rev.* **141**, 110790 (2021).
32. Lai, X. *et al.* Mechanism, modeling, detection, and prevention of the internal short circuit in lithium-ion batteries: Recent advances and perspectives. *Energy Storage Mater.* **35**, 470–499 (2021).
33. Attia, P. M. *et al.* Review—“Knees” in Lithium-Ion Battery Aging Trajectories. *J. Electrochem. Soc.* **169**, 060517 (2022).

34. Severson, K. A. *et al.* Data-driven prediction of battery cycle life before capacity degradation. *Nat. Energy* **4**, 383–391 (2019).

35. Harlow, J. E. *et al.* A Wide Range of Testing Results on an Excellent Lithium-Ion Cell Chemistry to be used as Benchmarks for New Battery Technologies. *J. Electrochem. Soc.* **166**, A3031 (2019).

36. Waldmann, T., Hogg, B.-I. & Wohlfahrt-Mehrens, M. Li plating as unwanted side reaction in commercial Li-ion cells – A review. *J. Power Sources* **384**, 107–124 (2018).

37. Preger, Y. *et al.* Degradation of Commercial Lithium-Ion Cells as a Function of Chemistry and Cycling Conditions. *J. Electrochem. Soc.* **167**, 120532 (2020).

38. Waldmann, T., Wilka, M., Kasper, M., Fleischhammer, M. & Wohlfahrt-Mehrens, M. Temperature dependent ageing mechanisms in Lithium-ion batteries – A Post-Mortem study. *J. Power Sources* **262**, 129–135 (2014).

39. Brand, M. J. *et al.* Effects of vibrations and shocks on lithium-ion cells. *J. Power Sources* **288**, 62–69 (2015).

40. Svens, P., Kjell, M., Tengstedt, C., Flodberg, G. & Lindbergh, G. Li-Ion Pouch Cells for Vehicle Applications — Studies of Water Transmission and Packing Materials. *Energies* **6**, 400–410 (2013).

41. Maddipatla, S., Kong, L. & Pecht, M. Electrolyte Leakage in Cylindrical Lithium-Ion Batteries Subjected to Temperature Cycling. *Energies* **17**, 1533 (2024).

42. Exacom - Battery Manufacturing Europe Solutions/Service Company. *Energy Business Review* https://web.archive.org/web/20240813011813/https://www.energybusinessreview.com/exacom (2024).

43. Najman, L. New Updates: How Long Do Electric Car Batteries Last? *Recurrent Auto* https://www.recurrentauto.com/research/how-long-do-ev-batteries-last (2024).

44. Witt, J. & Najman, L. Electric Car Battery Replacement Costs. *Recurrent Auto*

https://www.recurrentauto.com/research/costs-ev-battery-replacement (2024).

45. Tesla Model 3 Battery Size / Capacity & Range (new vs used). *Recurrent Auto* https://www.recurrentauto.com/guides/tesla-model-3.
